# Mitsugumin 53 protects the kidney from severe burn injury in mice

**DOI:** 10.4103/2321-3868.123074

**Published:** 2013-12-18

**Authors:** Yanjun Wu, Jian Huang, Daisong Liu, Jianglin Tan, Yanmeng Peng, Junjie Yang, Yanyan Cui, Weifeng He, Gaoxing Luo, Jun Wu

**Affiliations:** 1 Department of Urinary Surgery, Chengdu Seventh People’s Hospital, Chengdu tumor hospital, Chengdu, China; 2 Institute of Burn Research, Southwest Hospital, Third Military Medical University, Chongqing, China; 3 Institute of Burn Research, Southwest Hospital, Third Military Medical University, Chongqing, 400038 China

**Keywords:** Burn injury, recombinant human Mitsugumin53, kidney, polymerase I and transcript release factor, kidney injury molecule-1

## Abstract

Mitsugumin 53 (MG53), a newly identified muscle-specific protein, is an essential component of the cell membrane repair machinery in skeletal and cardiac muscle. However, the role of MG53 after burns in other tissues remains unclear. This study aims to investigate the possible roles of MG53 in the protection of the kidney after severe burn injury, and an animal scalding model of 30% of total body surface area (TBSA) was used. Recombinant human MG53 (rhMG53) or bovine serum albumin (BSA) was injected intravenously via the tail vein. Data showed that the mortality in the MG53-treated group was lower than that in control group. Administration of rhMG53 may alleviate histological alterations in renal tubular epithelial cells after burn injury. Renal tubular injury scores and the average optical density score of kidney injury molecule-1 (KIM-1) immunohistochemical staining in the MG53-treated group were significantly lower than those in control group (*P* < 0.001). Exogenous rhMG53 was found to be located in renal tubular epithelial cells. Numerous polymerase I and transcript release factor (PTRF) were expressed in the mouse kidney after severe scalding. In conclusion, our data indicate that MG53 protein protects the kidney by involving local PTRF after severe burn injury.

## Introduction

Internal organs participate in and sustain all types of activity for life. Many factors, such as acute ischemia, anoxia, and inflammatory factors, may induce multi-tissue damage in some internal organs after severe burn injury. Tissue damage in these internal organs has proven to be responsible for aggravation of disease, the development of complications, and increased mortality. Better prevention and timely rehabilitation and reconstruction of those important internal organs are critical for improving survival and reducing mortality. After severe scalding, the ischemic injury that occurs in the organs and the repair process does not attract public attention due to the lack of macroscopic evidence, the wide range of effects, the number of organs involved, its complex/concealed pathogenesis, and the ineffective repair-promoting measures used.[1] In the past, various measures were taken that targeted certain stages of pathogenesis or alleviated the degree of injury; however, they did not repair cells directly. Thus, they did not contribute to the initiation of organ repair. Therefore, investigating how to accelerate and enhance the initial repair of injured organs has become critical for successfully rescuing severely scalded patients and to prevent/treat the multiple organ dysfunction syndrome (MODS) and multiple system organ failure (MSOF) that occur later. It is an important research topic in medicine and is of great theoretical/clinical significance.

**Table 1:** 

Access this article online	
**Quick Response Code:**	**Website:** www.burnstrauma.com
	**DOI:** 10.4103/2321-3868.123074

Mitsugumin 53 (MG53), a muscle-specific tripartite motif (TRIM) family protein, is an essential component of the cell membrane repair machinery. Ma *et al.*, reported that recombinant human MG53 (rhMG53) may show therapeutic action against Duchenne muscular dystrophy as well as other human diseases that involve membrane injuries. The rhMG53 protein can recognize the external signals at the injured membrane sites and increase membrane resealing in cultured muscle and non-muscle cells when added to the culture medium. Animal studies have shown that application of rhMG53 via multiple routes (intramuscular, intravenous, or subcutaneous) improves the membrane repair capacity of skeletal myocytes and ameliorated muscular dystrophy.[2]

Therefore, it is reasonable to propose that exogenous rhMG53 may promote the recovery of damaged cells and repair of injured cell membranes in a burn model. Here, we report the possible roles of rhMG53 in the prevention of internal organ tissue injury and that it may reduce mortality in a mouse severe burn model.

## Materials and Methods

### Animals

Male BALB/c mice, weighing between 19 and 21 g, were provided by the Animal Center of the Third Military Medical University of China. These mice were maintained behind a barrier and were provided with plenty of food and water.

### Reagents

Kidney injury molecule-1 (KIM-1) antibody was purchased from Abcam, Cambridge, England). MG53 antibody was presented by Professor Ma Jianji, the Laboratory of UMDNJ-Robert Wood Johnson Medical School, Newark, New Jersey, USA. Polymerase I and transcript release factor (PTRF) antibodies were purchased from Proteintech Group, Chicago, Illinois, USA. Antigen retrieval buffers were purchased from Wuhan Boster Bio-engineering Co. Ltd. Goat serum, goat anti-rabbit secondary antibody, and horseradish peroxidase-labeled streptavidin were purchased from Beijing Zhong Shan Golden Bridge Bio-technology Co. Ltd.

### Burn model

Briefly, the dorsal part of the mouse was shaved. Mice were anesthetized with 1% amobarbital at a dose of 40 mg/kg, and the shaved part of the skin was burnt using a constanttemperature/pressure electric scalding device at 90°C for 8 s with 0.5 kg pressure, which induced a 30% total body surface area (TBSA) and full thickness burn injury, which was confirmed histologically. Immediately after the burn, a total of 2.5 ml of lactate-linger solution was injected intraperitoneally to treat for shock.[3,4]

### Test groups and method of administration

A total of 16 severely scalded mice were divided randomly into control group and MG53-treated group (MG53 group) with eight mice in each group. For the rhMG53-treated group, rhMG53 was injected twice into the tail vein at 0.5 and 6.5 h after scalding at 3 mg/kg,[2] and for the control group, a dose of bovine serum albumin (BSA) was administered.

### Mortality evaluation

The mortality of mice was recorded every 6 h within the 48 h after scalding.

### Tissue sample preparation and histopathological observation

At 48 h after the scalding, mice were anesthetized with 1% amobarbital and perfused via the left ventricle with 20 ml of a 4% paraformaldehyde solution. The heart, liver, spleen, lung, kidney, stomach, small intestine, colon, skin, muscle, and brain were then removed. All tissues were fixed in 4% paraformaldehyde solution, followed by dehydration, hyalinization, paraffin-embedding, and sectioning at 5 µm. All sections were stained with hematoxylin and eosin (HE) and observed under a microscope.

### Pathological scoring of the renal tubule for degree of necrosis

To measure the pathological score, 20 visual fields from each section were selected randomly under the microscope and were scored using a semiquantitative pathological assessment method (Erdogan *et al.*,) based on the degree of necrosis of the renal tubules.[5] Briefly, 0 denotes normal kidney morphology, 1 denotes rare necrosis (necrosis of <5% of renal tubules), 2 denotes mild necrosis (necrosis of 5–25% of renal tubules), 3 denotes moderate necrosis (necrosis of 25–75% of renal tubules), and 4 denotes severe necrosis (necrosis of >75% of renal tubules).

### Immunohistochemical staining

Antibodies targeted against KIM-1, MG53, and PTRF were obtained. Briefly, kidney paraffin-embedded sections were deparaffinized and then hydrated. The sections were then incubated with 3% H_2_O_2_ to deactivate endogenous peroxidases, followed by antigen retrieval, blocking with normal goat serum, and incubation with primary antibodies as follows: 1:100 KIM-1[6] polyclonal antibody, 1:100 rabbit anti-human MG53 antibody,[7] and 1:100 rabbit anti-human PTRF antibody. Sections were then incubated with goat anti-rabbit secondary antibody followed by horseradish peroxidase-labeled streptavidin. The sections were developed, stained with hematoxylin and observed under a microscope. Five fields were selected from each section at random, and photomicrographs were taken and analyzed semiquantitatively using Integrated Performance Primitives (IPP) software.

### Statistical analyses

Results are expressed as the mean ± standard deviation $\left( {\bar x \pm {\rm{sd}}} \right)$ and were analyzed using *t*-tests, Chi-squared tests, and Kaplan-Meier tests using Statistical Package for Social Sciences (SPSS) software and Microsoft Excel. A *P*-value of <0.05 was considered statistically significant.

## Results

### The role of rhMG53 in mortality of burn mice

**Figure 1:**
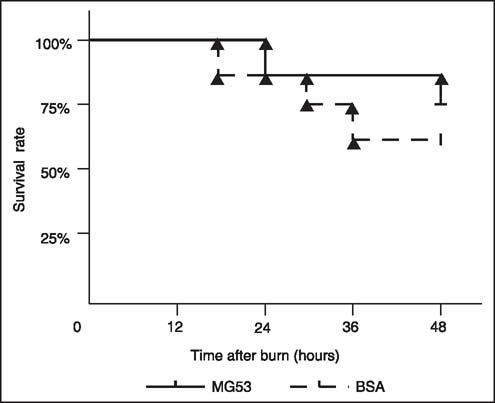
Survival curve of Mitsugumin 53 (MG53)-treated and bovine serum albumin (BSA)-treated mice in 48 h after severe burn injury. The mortality of the MG53 group was lower than that of the BSA group. However, there was no statistically significant difference between the two groups (*P* = 0.681).

The mortality at 24 h after burn injury was 12.5% in the MG53-treated group and 12.5% in the control group. At 48 h after burn injury, 25% of the MG53-treated group and 37.5% of the control group had died. However, there was no significant difference between the two groups at the time intervals tested although the MG53-treated group showed slightly less mortality than the control group [Figure 1] (*P* = 0.681).

### The effect of rhMG53 on histological alterations in internal organs

**Figure 2:**
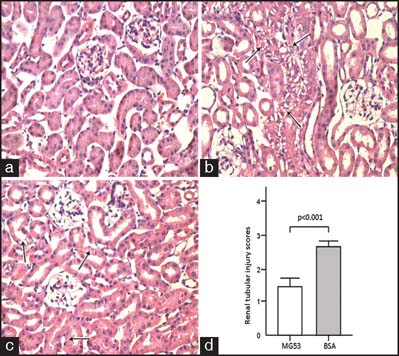
Kidney histology in tissue from mice in either the MG53- or BSA-treated group (hematoxylin and eosin (HE) staining, ×400). (a) Healthy renal tissue. (b) Example of renal tissue from the BSA-treated group. Vacuolar degeneration and necrosis of kidney tubules was common (indicated by the arrow). (c) Example of kidney tissue from the MG53-treated group. These injuries were less severe than those observed in the BSA-treated group (indicated by the arrow). Scale bar denotes 100 µm. (d) Renal tissue damage scores of each group. The score of the MG53 group was 1.43 ± 0.19. The score of the BSA group was 2.67 ± 0.14 (*P* < 0.001).

In the MG53-treated group, the epithelial cells of the proximal and distal convoluted renal tubules were improved significantly with respect to cloudy swelling and degeneration. In addition, less inflammatory cells were present in the renal mesenchyma in the MG53-treated group than in the control group. However, there was no significant improvement in renal glomerular area [Figure 2a, b, and c]. Moreover, the semiquantitative pathological score of the MG53-treated group was significantly lower than that of the control group [Figure 2d] (*P* < 0.001) at 48 h after scalding. However, HE-stained tissue sections from the heart, liver, spleen, lung, stomach, small intestine, colon, skin, muscle, and brain did not show differences between the MG53-treated group and control group.

### The expression of both KIM-1 and PTRF in kidney

**Figure 3:**
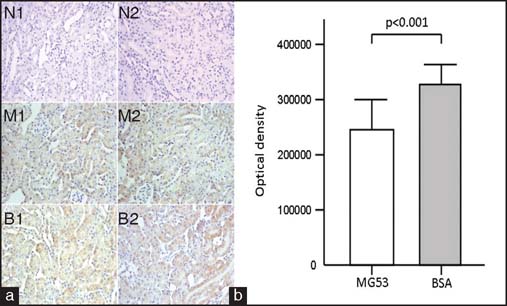
Expression of kidney injury molecule (KIM-1) in kidney of mice in each group. (a) KIM-1 immunohistochemical staining of mouse renal tissues from each groups (×400). N1–N2 = Negative control group, M1–M2 = MG53-treated group, B1–B2 = BSA-treated group. Scale bar denotes 100 µm. (b) KIM-1 protein expression in each group. The mean optical density score in the MG53-treated group was significantly lower than that of the BSA-treated group (240,190.9 ± 58,277.4 vs 342,880.3 ± 36,821.7, *P* < 0.001).

**Figure 4:**
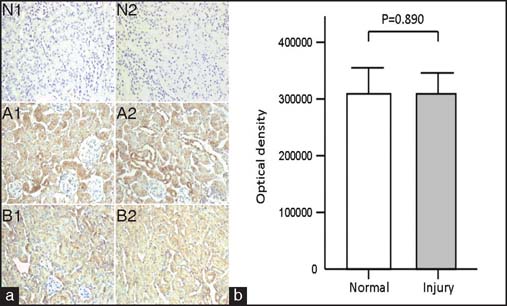
Expression of Polymerase I and transcript release factor (PTRF) in mouse kidney in each group. (a) PTRF immunohistochemical staining of mouse renal tissues in each group (×400). N1–N2 = Negative control group, A1–A2 = Control kidney. B1–B2 = MG53-treated kidney. Scale bar denotes 100 µm. (b) The mean optical density score for PTRF expression in each group. The score for control kidney was 313,110.9 ± 44,570.2. The score for MG53-treated kidney was 315,946.8 ± 35,043.3. There was no statistically significant difference in mean optical density score between the groups (*P* = 0.890).

KIM-1 positive cells were noted in the renal tissues of both groups [Figure 3a]; however, the average optical density score in the MG53-treated group was significantly lower than that in the control group [Figure 3b] (*P* < 0.001). PTRF-positive cells were observed in the renal tissues of both groups [Figure 4a]. No significant difference in mean optical density score was observed between the MG53-treated and control groups [Figure 4b] (*P* = 0.890).

### The distribution of exogenous rhMG53 in internal organs

**Figure 5:**
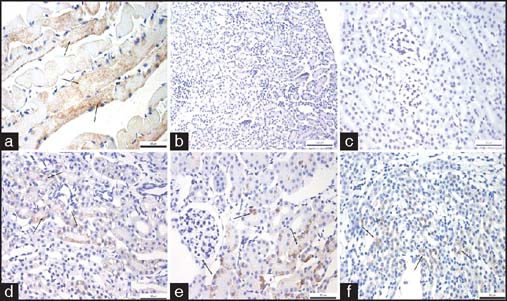
Expression of kidney injury molecule-1 (KIM-1) in mouse kidney in each group. MG53 immunohistochemical staining in mouse kidney tissues from each group (×400). (a) Endogenous MG53 is expressed in control muscle. (b) There is no MG53 expression in renal tissues. (c) Exogenous rhMG53 was not detected in renal tissue in the BSA-treated group. (d) Exogenous rhMG53 was detected in epithelial cells located in the (e) renal cortex and (f) renal medulla. Positive cells are indicated by the arrow. Scale bar denotes 100 µm.

Based on the immunohistochemical data, numerous MG53^+^ cells were noted in the cardiac/skeletal muscles of both groups but not in other organs of the control group. In the treatment group, rhMG53 was primarily distributed in a limited number of epithelial cells of the renal tubule. [Figure 5].

## Discussion

Scalding not only injures local tissues at the wound site, but also causes systemic reactions/injury to a certain extent. After scalding, acute ischemia/anoxia occurs in the organs for various reasons, e.g., early stress reactions, changes in vascular permeability, microcirculation disorders, and water/electrolyte/acid-base imbalance.[8]

The kidney is quite sensitive to ischemia/anoxia. After severe scalding, body fluid is lost rapidly, which causes acute renal ischemia/anoxia and a series of significant renal morphological changes.[9] The epithelial cells of the renal tubule are very vulnerable to ischemia/anoxia injury. Due to scald stress and early shock, the renal artery becomes ischemic, and the blood flow redistributes in the kidney, causing ischemia/anoxia in renal histocytes (in particular, some cells of the renal cortex), which further damages the cell membrane and finally causes irreversible injury/death. The necrosis of indigenous renal cells leads to a drop in the number of functional renal units, further affecting kidney function.

As shown in recent research, the injured cytoplasmic membrane can be repaired via a complex, multiple-step active dynamic reaction process that is mediated by MG53.[7] MG53 is one of the unique muscle TRIM-family proteins, and it is expressed only in cardiac/skeletal muscles. It is an essential component of the repair mechanism for membrane injury[10] After membrane injury, MG53 receives the initial signal of membrane injury (i.e., the exposure of membrane lipid molecules).[7,11] Mediated by PTRF, MG53 is then transferred to near the injured membrane in cellular vesicles and is bound to the exposed membrane lipid molecules.[12,13] Third, the vesicles merge with the cytoplasmic membrane to establish the repair process.[14,15] As also shown by previous data, acute membrane-damage signals can be detected by environmental MG53 proteins that then repair the injured cell membrane.[2] However, MG53 cannot bind the exposed membrane lipid molecule by itself; thus, one mediating molecule is required at the injury site to anchor MG53 to the exposed lipid. PTRF (also called “cavin-1”) is a docking protein that binds MG53 at the exposed membrane lipid at the injury site during MG53-mediated membrane repair. PTRF is expressed in most organs (except the liver) including the kidney, lung, heart, and the skeletal muscles, among others.[12]

In our mouse model, we observed kidney-specific distribution of exogenous rhMG53. After burn injury, the exogenous rhMG53 significantly alleviated damage to renal tissue, but not that to other internal tissues. At 48 h after damage, histopathological analysis revealed milder renal injury and lower scores for renal tubule lesions in the MG53-treated group compared with the control group. KIM-1 immunohistochemical results also revealed a protective effect of exogenous MG53 in the kidney as KIM-1 expression in the MG53-treated group was significantly lower than in the BSA-treated group. Thus, we believe that after tail vein injection, exogenous rhMG53 arrived selectively at the site of injury in the kidney, in particular, the epithelial cells of the renal tubule, and repaired the injury.

Based on immunohistochemistry, we noted that exogenous rhMG53 was distributed in mouse kidney (primarily in the epithelial cells of the renal tubule at the subcapsule area and cortex-medulla interface), and the histopathological characteristics of the tissue were significantly improved compared with the control group. Zhu *et al.*, demonstrated that PTRF was expressed in mouse kidney, lung, heart, and skeletal muscles, and MG53 did not aggregate or mediate repair in the injured cells not expressing the endogenous PTRF[12] Based on our immunohistochemistry results, we noted numerous PTRF^+^ cells in the kidney of the injured group and there was no distinct difference between the injured and control group. Exogenous rhMG53 protein distributed in the kidney to areas of high PTRF expression. However, exogenous rhMG53 was not distributed significantly in other tissues that expressed less or no endogenous PTRF. All of the above results indicate that the selective distribution and repair mediated by exogenous MG53 correlated closely with PTRF.

After severe burn injury, many factors can induce multitissue damage in some internal organs. As the basic functional unit of the organism, cells degenerate when the organ is damaged, and the cell membrane is the initial site of damage. MG53 is an acute membrane damage-repair protein. MG53 can reach the membrane damage site quickly with other cofactors after acute damage to complete the injury repair. Exogenous rhMG53 protein injected into the tail vein selectively arrives at tissues where PTRF is highly expressed. Through PTRF and other cofactors, MG53 can repair the membrane damage and reduce organ injury.

In conclusion, for the first time, we show that exogenous rhMG53 can selectively be distributed to sites in the kidney that show high expression of PTRF, thereby ameliorating histological alterations in renal tissues after severe burn injury. Our data provide a novel mechanism for protecting the kidney from injury that may be important for future treatment of burns in the clinic.
